# Specific Alternation of Gut Microbiota and the Role of *Ruminococcus gnavus* in the Development of Diabetic Nephropathy

**DOI:** 10.4014/jmb.2310.10028

**Published:** 2023-12-26

**Authors:** Jinni Hong, Tingting Fu, Weizhen Liu, Yu Du, Junmin Bu, Guojian Wei, Miao Yu, Yanshan Lin, Cunyun Min, Datao Lin

**Affiliations:** 1 Department of Traditional Chinese Medicine, Guangdong Provincial People's Hospital (Guangdong Academy of Medical Sciences), Southern Medical University, Guangzhou, 510080, P.R. China; 2 Guangdong Provincial Institute of Geriatric, Guangzhou, 510080, P.R. China; 3 Department of Parasitology, Zhongshan School of Medicine, Sun Yat-sen University, Guangzhou, 510080, P.R. China

**Keywords:** Gut microbiota, diabetic nephropathy, *Ruminococcus gnavus*, inflammation, uremic toxins

## Abstract

In this study, we aim to investigate the precise alterations in the gut microbiota during the onset and advancement of diabetic nephropathy (DN) and examine the impact of *Ruminococcus gnavus* (*R. gnavus*) on DN. Eight-week-old male KK-Ay mice were administered antibiotic cocktails for a duration of two weeks, followed by oral administration of *R. gnavus* for an additional eight weeks. Our study revealed significant changes in the gut microbiota during both the initiation and progression of DN. Specifically, we observed a notable increase in the abundance of Clostridia at the class level, higher levels of Lachnospirales and Oscillospirales at the order level, and a marked decrease in Clostridia_UCG-014 in DN group. Additionally, there was a significant increase in the abundance of Lachnospiraceae, Oscillospiraceae, and Ruminococcaceae at the family level. Moreover, oral administration of *R. gnavus* effectively aggravated kidney pathology in DN mice, accompanied by elevated levels of urea nitrogen (UN), creatinine (Cr), and urine protein. Furthermore, *R. gnavus* administration resulted in down-regulation of tight junction proteins such as Claudin-1, Occludin, and ZO-1, as well as increased levels of uremic toxins in urine and serum samples. Additionally, our study demonstrated that orally administered *R. gnavus* up-regulated the expression of inflammatory factors, including nucleotide-binding oligomerization domain-like receptor pyrin domain-containing protein 3 (NLRP3) and Interleukin (IL)-6. These changes indicated the involvement of the gut-kidney axis in DN, and *R. gnavus* may worsen diabetic nephropathy by affecting uremic toxin levels and promoting inflammation in DN.

## Introduction

Diabetic nephropathy (DN) is the leading cause of end-stage renal disease (ESRD), accounting for a significant proportion of cases worldwide. Approximately 21.8% to 40% of individuals with diabetes are affected by DN [1]. This disease carries a high burden of disability and mortality, posing a major public health threat. Despite the availability of various treatment options, the incidence of DN continues to rise.

The underlying mechanisms of DN remain poorly understood, which greatly hinders prevention and early detection efforts [2, 3]. Recently, much attention has been paid to the role of the gut microbiota, which not only plays a crucial part in maintaining intestinal balance but also contributes to the development of metabolic diseases like obesity, diabetes mellitus (DM), and chronic kidney disease (CKD) [4-7]. Noticeable changes in the composition of the gut microbiome in DM patients were observed, characterized by a decrease in beneficial butyrate-producing bacteria and an increase in harmful pathogens [8]. Imbalances in the gut microbiota, including an increase in Proteobacteria, Verrucomicrobia, and Fusobacteria, have been observed in patients with DN [9]. An intimate connection between gut and kidney has been proposed [10]. Gut microbiota dysbiosis leads to the production of abnormal metabolites and compromises the integrity of the intestinal barrier, potentially damaging the kidney and other organs by affecting insulin sensitivity, glucose metabolism, and immune function [11]. Manipulating the gut microbiota can partially improve renal injury associated with diabetes by reducing oxidative stress and inflammation [12]. Specific metabolites and toxins produced by the gut microbiota, such as trimethylamine-N-oxide (TMAO), p-cresyl sulfate (pCS) and indoxyl sulfate (IS) have been implicated in DN pathology [13, 14]. Increasing the abundance of short-chain fatty acids (SCFAs) -producing bacteria can help regulate intestinal inflammation, improve host immunity, and even influence insulin sensitivity and energy metabolism, thus inhibiting disease progression [15, 16]. Therefore, the gut microbiota and derived metabolites hold promise as potential targets for therapeutic interventions in DN. However, the precise molecular mechanisms through which the gut microbiota contributes to the development of DN are still unclear.

Among the 57 most common species of the human gut microbiome, there is a specific bacterium called *Ruminococcus gnavus* that has consistently been associated with various conditions such as inflammatory bowel disease (IBD), obesity, DM, neurological disorders, and cancer [17]. Recently, two large-scale cohort studies demonstrated a positive correlation between *R. gnavus* and body fat percentage in patients with metabolic syndrome [18]. Furthermore, evidence suggests that an imbalanced representation of *R. gnavus*, either decreased or increased, can affect the pathology of certain diseases such as atopic dermatitis and IBD [19, 20]. However, it remains unclear whether *R. gnavus* actively participates in disease pathogenesis or merely expands due to a competitive advantage during inflammation. There is still a lack of research studying the regulatory mechanisms governing *R. gnavus* in DN.

Inflammation is involved throughout the entire process of DN and is a focal point of current research on the pathogenesis of DN [21]. The theory of metabolic inflammation was first proposed in 2006, which posits that the accumulation of nutrients and metabolic byproducts can provoke a chronic low-grade inflammatory response, consequently contributing to the onset and progression of metabolic disorders [22]. DN is a form of metabolic inflammation, where chronic inflammatory reactions can directly induce morphological and functional changes in renal intrinsic cells, resulting in inflammatory kidney damage [23]. Inflammation is not only a key factor in the progression of DN, but also a potential therapeutic target [23]. Compared to healthy individuals, patients with DM and DN show increased expression of inflammatory factors such as interleukin (IL)-6 and tumor necrosis factor-alpha (TNF-α) in their serum [23]. In DN mice, the levels of advanced glycation end-products (AGEs) and their receptors are upregulated, and activated nucleotide-binding oligomerization domain-like receptor pyrin domain-containing protein 3 (NLRP3) inflammasome, contribute to the progression of the disease [24].

In this study, we aimed to explore the characteristics of the gut microbiota during the progression of DN. Subsequently, we investigated the effects of orally administering *R. gnavus* in a mouse model of DN, assessing its impact on inflammation and uremic toxin-related mechanisms. By shedding light on the role of *R. gnavus* and its potential therapeutic implications for DN, this study aimed to contribute to a better understanding of DN.

## Materials and Methods

### Animal Grouping and Treatment

At the age of 12 weeks old, KK-Ay mice can develop early renal damage characterized by increased glomerular area, thickened glomerular basement membrane, mesangial matrix proliferation, and sclerotic nodules. These pathological changes observed in mice resemble the early stages of DN in humans [25, 26]. As a result, KK-Ay mice are commonly used in early DN research.

All animal experiments were performed in accordance with the guidelines of the NIH for the care and use of laboratory animals. The study was conducted with the approval of the Institutional Animal Care and Use Committee of Guangdong Provincial Peoplés Hospital (Approval Number: KY2023-018-01). Male KK-Ay mice (N=25) and C57BL/6J mice (N=5) were obtained from Beijing HFK Bioscience Co., Ltd. [License No. SCXK (Jing) 2019-0008]. The mice were eight weeks old at the start of the experiment. KK-Ay mice were fed a sterile high-fat diet to induce the DN model, while C57BL/6J mice were fed a sterile regular diet. They were housed in a specific pathogen-free (SPF) environment at a temperature of 22 ± 1°C, with a 12-h light-dark cycle.

After a one-week acclimation period, the mice were randomly divided into the following six groups: (1) C57 (C57BL/6J mice treated with sterile saline solution); (2) KK (KK-Ay mice treated with sterile saline solution); (3) anti (KK-Ay mice treated with antibiotic intervention); (4) low (KK-Ay mice treated with antibiotic intervention followed with 10^7^ CFU *R. gnavus*); (5) mid (KK-Ay mice treated with antibiotic intervention followed with 10^8^ CFU *R. gnavus*); and (6) high (KK-Ay mice treated with antibiotic intervention followed with 10^9^ CFU *R. gnavus*). To induce the antibiotic-treated model, the mice in the antibiotic-treated group received a daily oral administration of an antibiotic mix containing Vancomycin (50 mg/kg), Neomycin (100 mg/kg), and Metronidazole (100 mg/kg). Additionally, they were exposed to Amoxicillin (1 mg/ml) in their drinking water. These treatments were carried out for a duration of two weeks.

**Fig. 1:**
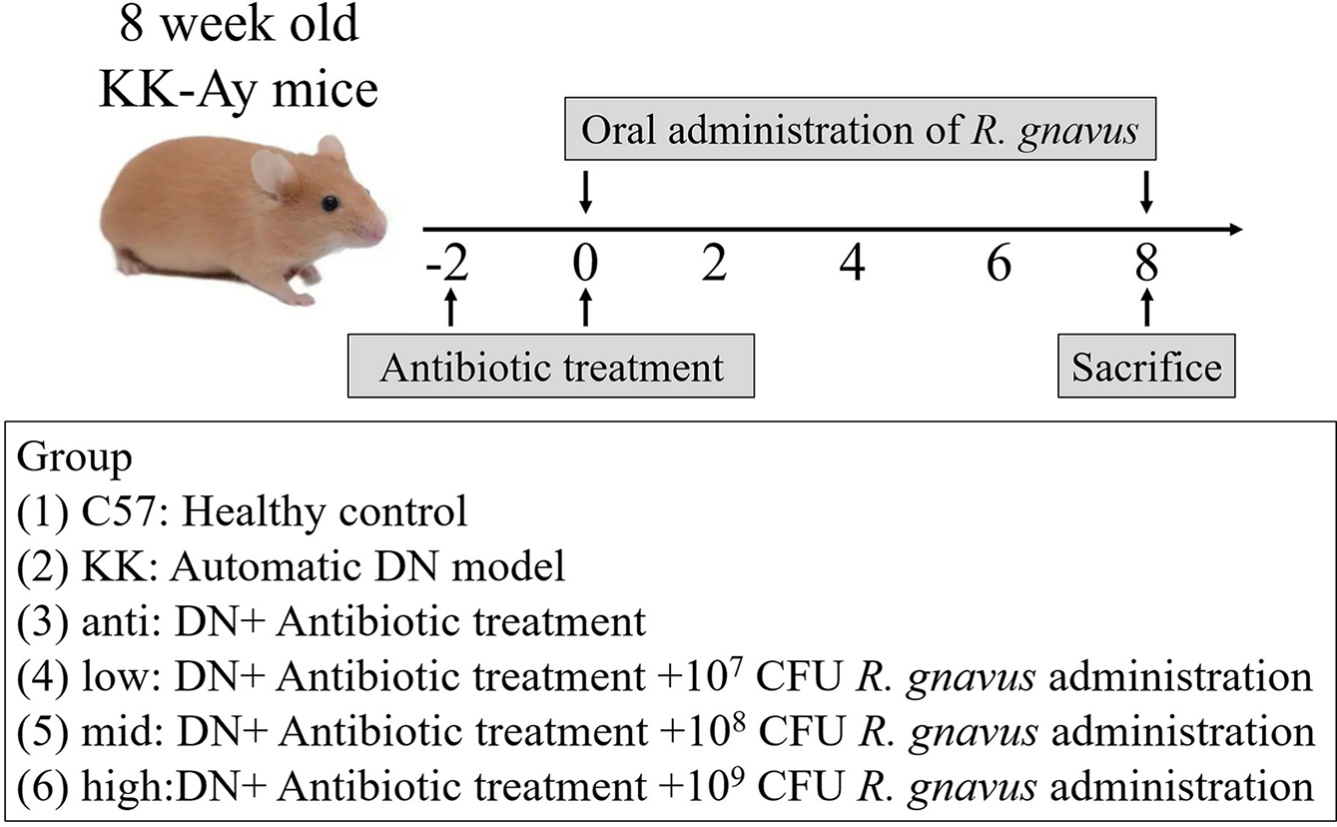
Experimental protocol for examining the effects of *R. gnavus* in KK-Ay mice.

The strain of *R. gnavus* ATCC 29149 was obtained from the American Type Culture Collection (ATCC). It was cultured in peptone yeast glucose (PYG) under anaerobic conditions at 37°C, overnight. Then, bacteria in optical density (OD)600=1 culture were washed once with sterile phosphate buffered saline (PBS; pH 7.4) and re-suspended in sterile saline solution to achieve a final concentration of 5 × 10^8^ CFU/ml. The mice in the low, mid, and high groups received oral administration of *R. gnavus*, the concentration was determined basing on previous studies [27, 28], while the C57 and KK groups were given sterile saline solution. The administration of *R. gnavus* or saline solution continued for eight weeks until the endpoint of the study. The protocol was depicted in Fig. 1.

### Fecal Microbiota Analysis

**Sample collection, DNA extraction and PCR amplification.** Fecal samples were collected from the mice before treatment and after two, four, and eight weeks’ treatment. They were individually collected and subjected to separate 16S rRNA gene sequencing. The data from the same group were averaged for inter-group comparisons. The mice were placed in metabolic cages for a 24-h period to enable fecal collection.

Genomic bacterial DNA was extracted from the fecal samples using the PF Mag-Bind Stool DNA Kit (Omega Bio-tek, USA). The quality and concentration of the extracted DNA were assessed using agarose gel electrophoresis and a NanoDrop ND-2000 spectrophotometer. The DNA samples were stored at -80°C until further use. The hypervariable region V3-V4 of the bacterial 16S rRNA gene were amplified with primer pairs 338F (5'-ACTCCTACGGGAGGCAGCAG-3') and 806R (5'-GGACTACHVGGGTWTCTAAT-3') [29] by an ABI GeneAmp 9700 PCR thermocycler (ABI, USA). The PCR reaction mixture including 4 μl 5×Fast Pfu buffer, 2 μl 2.5 mM dNTPs, 0.8 μl each primer (5 μM), 0.4 μl Fast Pfu polymerase, 0.2 μl BSA, 10 ng of template DNA, and ddH_2_O to a final volume of 20 μl. PCR amplification cycling conditions were as follows: initial denaturation at 95°C for 3 min, followed by 27 cycles of denaturing at 95°C for 30 s, annealing at 55°C for 30 s and extension at 72°C for 45 s, and single extension at 72°C for 10 min, and end at 4°C. Each sample was amplified in triplicate to reduce experimental errors. The PCR product was extracted from 2% agarose gel and purified. Then quantified using Quantus Fluorometer (Promega, USA).

**Illumina novaseq6000 sequencing and analysis.** The purified amplicons were combined in equal amounts and subjected to paired-end sequencing on an Illumina PE250 platform (Illumina, USA) following standard protocols. This allowed for high-throughput sequencing of the microbial DNA. After demultiplexing, the resulting sequences were quality filtered with fastp (0.19.6) [30] and merged with FLASH (v1.2.7) [31]. The resulting high-quality sequences were de-noised using the DADA2 [32] plugin in the Qiime2 (version 2022.2) pipeline [33]. This process generated amplicon sequence variants (ASVs), which are highly accurate representations of the microbial populations present in the samples. In order to account for any variations in sequencing depth, the number of sequences from each sample was rarefied to a standardized value of 4115.

The Majorbio Cloud platform (https://cloud.majorbio.com) was utilized for bioinformatic analysis of the fecal microbiota data. Based on the ASVs, various analyses were performed with Mothur (v1.30.2) [34], including the calculation of rarefaction curves and alpha diversity indices such as observed ASVs, Chao richness, Shannon index, and Good's coverage. Different visualization methods, including bar plots, pie charts, Circos plots and heatmap, were employed to analyze the diversity of the microbial communities. Statistical tests such as the Wilcoxon rank-sum test and Kruskal-Wallis H test were conducted to assess the differences in microbiota composition between two or more groups.

### Renal Function Analysis

Pre-treatment and at two, four and eight weeks after the initiation of treatment, the mice were placed in metabolic cages for 24-h for urine collection. After the eight-week treatment period, blood samples were collected from the micé caudal veins. The levels of urea nitrogen (UN), creatinine (Cr), and urine protein in the collected urine samples were measured using a commercial kit from Nanjingjiancheng Inc. (China). Additionally, the kidneys were removed and subjected to analysis using electron microscopy. The concentration of kidney injury marker-1 (KIM-1) in both urine and blood samples was determined using an ELISA kit provided by mmbio Inc.(China).

### Inflammatory Factors Measurement

Following the completion of a eight-week treatment period, serum samples were obtained. The concentrations of NLRP3 and IL-6 in the serum were determined using commercial ELISA kits sourced from ELK biotechnology (China), following the manufacturer's recommended protocol.

### Uremic Toxins Measurement

Prior to and at two, four and eight weeks into the treatment period, the mice were placed in metabolic cages for 24-h urine collection. After eight weeks of treatment, serum samples were collected from the mice. Commercial ELISA kits sourced from mmbio (China) were used to measure the concentrations of uremic toxins (TMAO, pCS, and IS) in both the urine and serum samples. The measurements were carried out following the protocols provided by the manufacturer.

### Immunohistochemistry Analysis

The colons were fixed in 4% paraformaldehyde for four hours and then transferred to 70% ethanol for preservation. Subsequently, the samples were sliced into four-μm-thick sections and embedded in paraffin. These colon sections were subjected to overnight incubation at 4°C with specific primary antibodies: Recombinant anti-Claudin-1 antibody (GB15032, Servicebio, China) at a dilution of 1:500 (Mouse mAb), anti-Occludin Rabbit pAb (GB111401, Servicebio) at a dilution of 1:500, or anti-ZO-1 tight junction protein Rabbit pAb (GB11195, Servicebio) at a dilution of 1:500. Following this, the sections were treated with a goat anti-rabbit secondary antibody (Beijing Zhong Shan Golden Bridge Biotechnology Co., Ltd., China) for one hour at room temperature. The sections were examined using an Olympus DY07 microscope (Olympus, Japan) and high-resolution images were captured using a camera at a magnification of 400.

### Ultra-Structural Analysis

After a eight-week treatment period, the renal cortexes of the mice were surgically removed and fixed in 2.5%glutaraldehyde at 4°C. Subsequently, they were embedded in epoxy resin for preservation. The ultramicrotome was utilized to cut thin sections from the embedded tissue, with a thickness ranging between 70 and 90 nm. To enhance contrast, the ultra thin sections were double-stained with 3% uranyl acetate and lead citrate. Finally, the sections were observed and analyzed using a JEM-1400 electron microscope (Jeol Ltd., Japan), allowing for detailed examination of the ultra-structural features.

### Statistical Analysis

All data were presented as mean ± standard deviation (SD). SPSS 19.0 (IBM, USA) was used to analyze data. The difference between the two groups was compared by using the student's *t*-test. The difference among multiple groups was compared by using the one-way analysis of variance (ANOVA) followed by the LSD or Tukey's post hoc test. Differences were considered statistically significant if *p* < 0.05.

## Results

### The Alternation of Gut Microbial Composition in DN

**Fig. 2:**
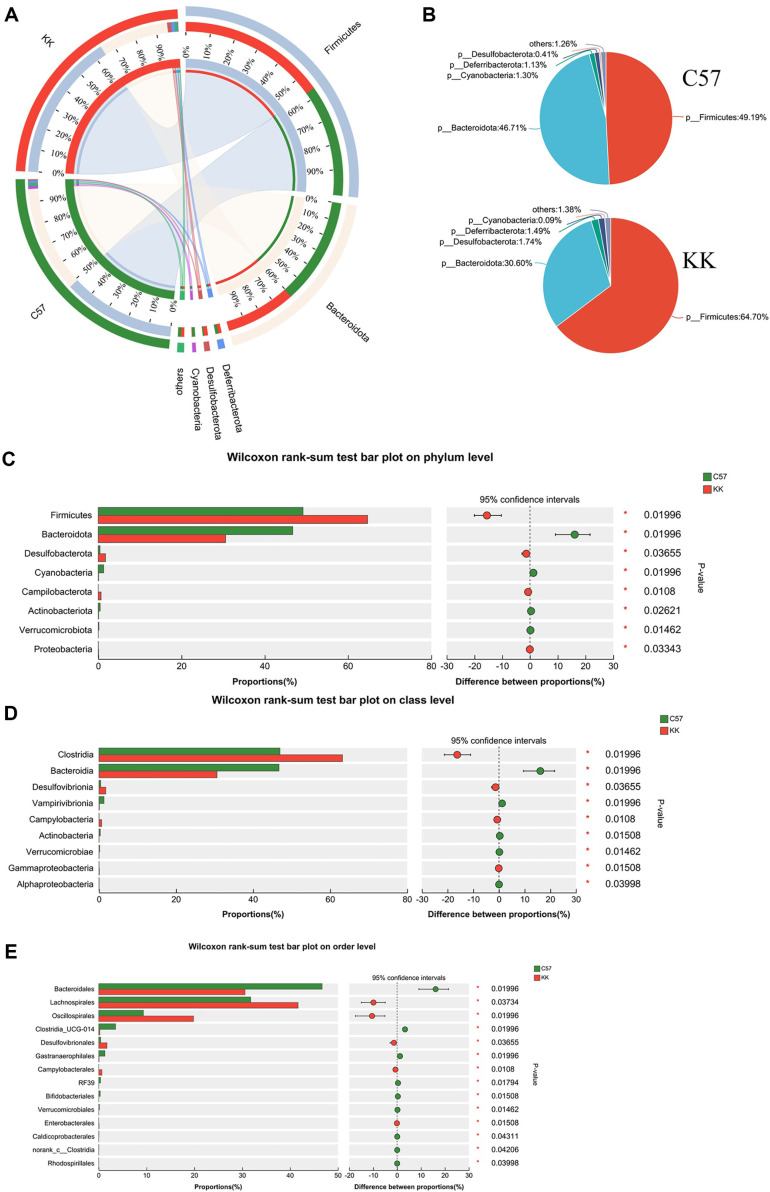
Gut microbiota compositions ranging from phylum to order levels in C57 and KK-Ay mice. (**A**) Circos analysis providing a visual representation of the gut microbiota composition at the phylum level; (**B**) Community analysis pie plot presenting the relative abundance of different phyla in the gut microbiota; (**C-E**) Wilcoxon rank-sum test bar plots comparing the phylum, class and order level gut microbiota composition, respectively. C57, C57BL/6J group; KK, KK-Ay group; *p < 0.05, v.s. C57BL/6J group.

In order to study the alterations in gut microbial composition in DN, we compared the fecal microbial differences between KK-Ay mice and C57 mice using 16S rRNA gene sequencing. At the phylum level in Fig. 2A and 2B, the Circos analysis and pie plot demonstrated that in C57 mice, the proportions of Firmicutes, Bacteroidota, Cyanobacteria, Deferribacterota, and Desulfobacterota were 49.19%, 46.71%, 1.30%, 1.13%, and 0.41% respectively. In KK-Ay mice, there was an increase in Firmicutes (64.70%), Deferribacterota (1.49%), and Desulfobacterota (1.74%), while Bacteroidota (30.60%) and Cyanobacteria (0.09%) showed a decrease. The Wilcoxon rank-sum test bar plot in Fig. 2C illustrated the significant differences at the phylum level, with increased levels of Firmicutes, Desulfobacterota, Campilobacterales, and Proteobacteria in KK-Ay mice compared to C57 mice. Conversely, Bacteroidota, Cyanobacteria, Actinobacteria and Verrucomicrobiota exhibited a decrease. At the class level, the bar plot in Fig. S1A indicated the increase of Clostridia and Desulfobacterota and lower abundance of Bacteroidia, Bacilli and Vampirivibrionia in KK-Ay mice when compared to C57. The heatmap in Fig. S1B indicated the changes of Vampirivibrionia, Campylobacteria, Actinobacteria, Coriobacteria, Verrucomicrobiae, Alphaproteobacteria, Negativicutes, Cyanobacteria, Saccharimonadia, Gammaproteobacteria, Bacilli, Deferribacteres, Desulfovibrionia, Clostridia and Bacteroidia. Wilxocon rank-sum test bar plot in Fig. 2D demonstrated significant higher abundance of Clostridia, Desulfovibrionia, Campylobacteria and Gamma-proteobacteria, and lower richness of Bacteroidia, Vampirivibrionia, Actinobacteria, Verrucomicrobiae and Alphaproteobacteria in KK-Ay mice than C57. Similarly, at the order level, the bar plot in Fig. S1C indicated the increase of Lachnospirales, Oscillospirales, Deferribacterales and Desulfovibrionales, as well as decreased Bacteroidales, Clostridia_UCG-014, Erysipelotrichales, Clostridia_vadinBB60_group and Gastranaerophilales in KK-Ay mice when compared to C57. The heatmap in Fig. S1D indicated the changes of Bacteroidales, Lachnospirales, Oscillospirales, Clostridia_UCG-014, Deferribacterales, Desulfovibrionales, Campylobacterales, RF39, Bifidobacteriales, Peptococcales, Peptostreptococcales-Tissierellales, Coriobacteriales, Verrucomicrobiales, Caldicoprobacterales, Staphylococcales, Veillonellales-Selenomonadales, Chloroplast, Christensenellales, Rhodospirillales, Acholeplasmatales, Bacillales, Saccharimonadales, Clostridiales, Enterobacterales, Gas-tranaerophilales, Erysipelotrichales and Lactobacillales. Wilxocon rank-sum test bar plot in Fig. 2E demonstrated significant higher abundance of Lachnospirales, Oscillospirales, Desulfovibrionales, Campylobacterales and Enterobacterales, lower abundance of Bacteroidales, Clostridia_UCG-014, Caldicoprobacterales, Verrucomicrobiales, Bifidobacteriales, RF39, Gastranaerophilales and Rhodospirillales in KK-Ay mice when compared to C57.

**Fig. 3:**
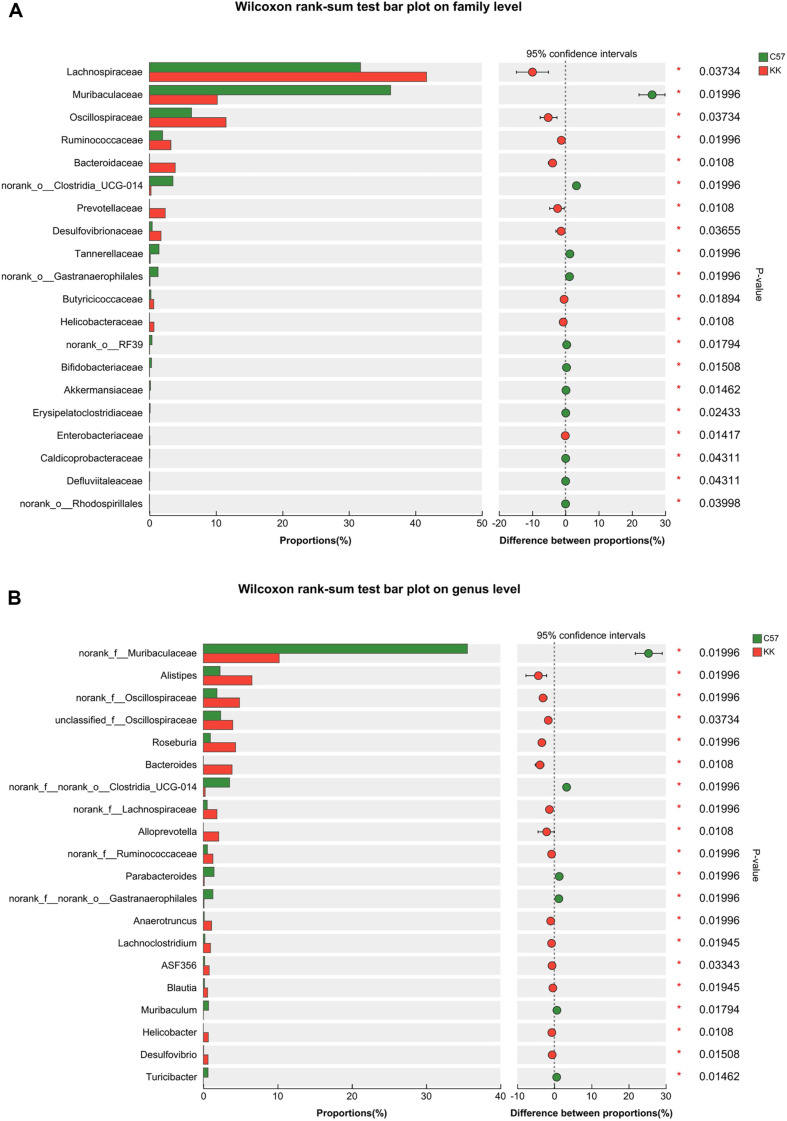
Wilcoxon rank-sum test bar plots at the family and genus levels in C57 and KK-Ay mice. (**A**) At the family level; (**B**) At the genus level. C57, C57BL/6J group; KK, KK-Ay group; *p < 0.05, v.s. C57BL/6J group.

At the family level, the Circos analysis in Fig. S2A and bar plot in Fig. S2B indicated an increase in the abundance of Lachnospiraceae, Oscillospiraceae, Rikenellaceae, Marinifilaceae, Ruminococcaceae, Eubacterium_ coprostanoligenes_group, Bacteroidaceae, Deferribacteraceae, Prevotellaceae, Desulfovibrionaceae, as well as a decrease in the abundance of Muribaculaceae, Tannerellaceae and Erysipelotrichaceae. The Wilxocon rank-sum test bar plot in Fig. 3A further demonstrated a significant increase in the abundance of Lachnospiraceae, Oscillospiraceae, Ruminococcaceae, Bacteroidaceae, Prevotellaceae, Desulfovibrionaceae, Butyricicoccaceae, Helicobacteraceae, and Enterobacteriaceae, as well as a decrease in the abundance of Muribaculaceae, Bifidobacteriaceae, Akkermansiaceae, Tannerellaceae, Defluviitaleaceae, Caldicoprobacteraceae, Erysipelato-clostridiaceae and Akkermansiaceae.

The bar plot in Fig. S2C and the heatmap in Fig. S2D indicated changes in microbial composition at the genus level, including *Lachnospiraceae_NK4A136_group*, *Bacteroides*, *Roseburia*, *Alistipes*, *Odoribacter*, *Rikenellaceae_RC9_gut_group*, *Parabacteroides*, *Anaerotruncus*, *Bilophila*, *Lachnoclostridium*, *ASF356Rikenella*, *BlautiaHelicobacter*, *Desulfovibrio*, *Lactobacillus*, *Lachnospiraceae_UCG-006*, *Butyricicoccus*, *A2*, *Acetatifactor*, *Intestinimonas*, *Eubacterium_oxidoreducens_group*, *Ruminococcus*, *Anaerostipes*, *MuribaculumTuricibacter*, *Alloprevotella*, *Lachnospiraceae_UCG-001*, *Oscillibacter*, *Mucispirillum*, and *Colidextribacter*. The Wilxocon rank-sum test in Fig. 3B further calculated the significant differences, showing a significantly higher abundance of Alistipes, Roseburia, Bacteroides, Anaerotruncus, Lachnoclostridium, ASF356, Blautia, Helicobacter, and Desulfovibrio, as well as a lower abundance of Parabacteroides, Muribaculum, and Turicibacter. Fig. S2E showed the Wilxocon rank-sum test bar plot analysis at the species level of C57 and KK-Ay mice.

### The Gut Microbial Diversity and Microbial Composition in KK-Ay Mice with Different Ages

KK-Ay mice can develop early renal damage at the age of 12 weeks. Therefore, in this study, we chose 10 week-old KK-Ay mice as model, and compared gut microbial diversity and microbial composition of KK-Ay mice after zero, two, four and eight weeks’ treatment, to be more specific, KK-Ay mice at the age of 10 weeks, 12 weeks, 14 weeks and 18 weeks were compared.

According to Fig. S3, the coverage was similar across all four groups. The fecal alpha-microbial richness, as measured by ACE, Chao, and Sobs indexes, increased with age. The diversity of the gut microbiome, as indicated by the community diversity calculated using the Shannon index, also increased with age, while the Simpson index showed a decrease.

**Fig. 4:**
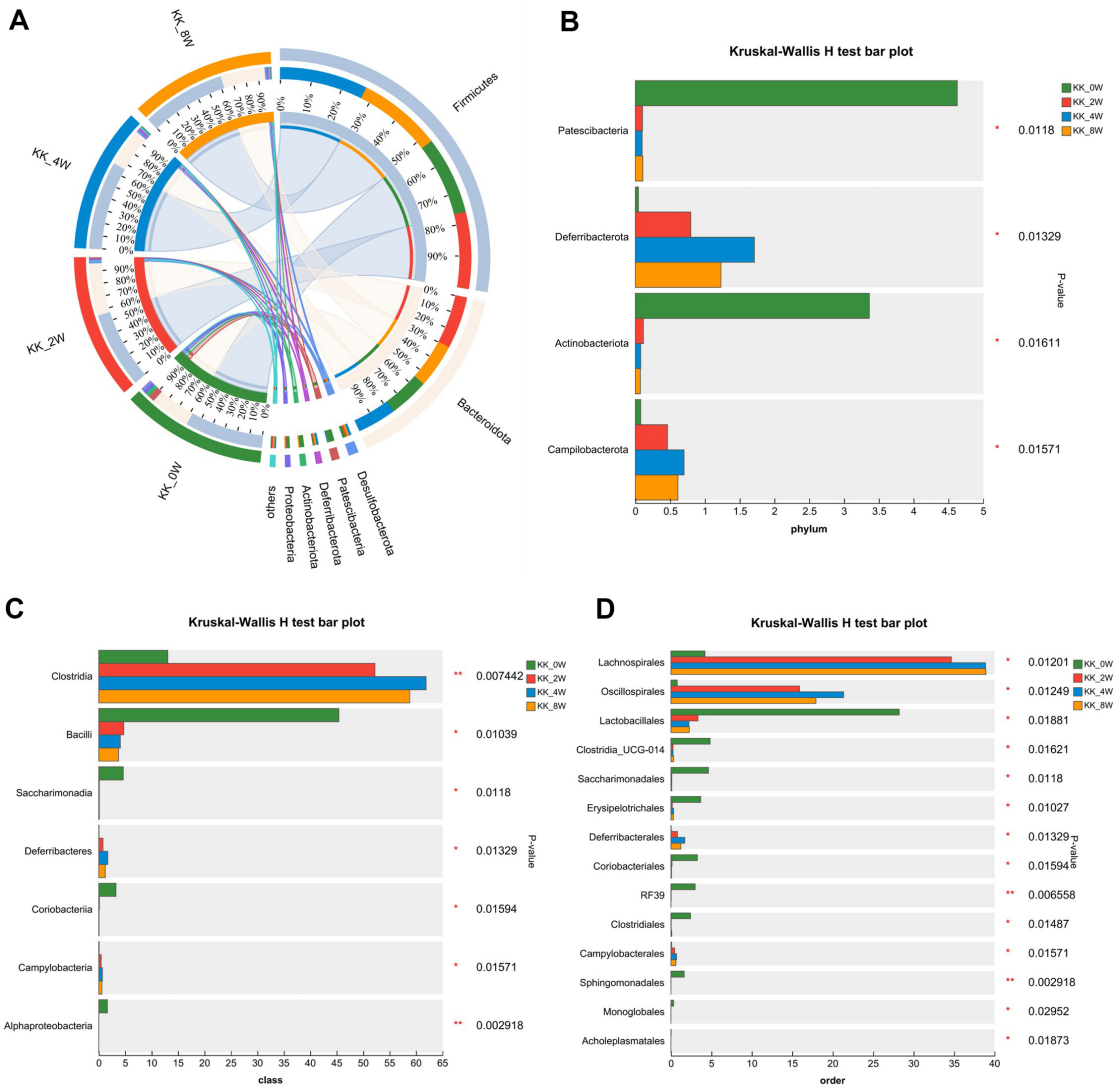
Gut microbiota compositions ranging from phylum to order of KK-Ay mice with different age. (**A**) The Circos analysis displays the gut microbiota composition at the phylum level; (**B-D**) The Kruskal-Wallis H test bar plots show the statistical significance of differences at the phylum, class and order level, respectively. KK_0W, KK-Ay mice at 10 weeks old; KK_2W, KK-Ay mice at 12 weeks old; KK_4W, KK-Ay mice at 14 weeks old; KK_8W, KK-Ay mice at 18 weeks old; *p < 0.05, between groups; **p < 0.01, between groups.

To identify specific bacterial taxa associated with the progress of DN, we compared fecal microbiome using Circos, bar plot and heatmap. The Circos in Fig. 4A and bar plot in Fig. S4A depicted the overall changes of microbiota at the phylum level. The Kruskal-Wallis H test showed that the relative abundances of Patescibacteria and Actinobacteriota were down-regulated as the age increased, with *p* = 0.0118 and 0.01611 respectively. Compare to KK_0W, the relative abundances of Deferribacterota increased in KK_2W and KK_4W group. And it decreased at KK_8W, when compared to KK_4W group, with a significant difference. Similar tendency was found in Campilobacterota, with significant difference (*p* = 0.01571, Fig. 4B). At the class level. As shown in Fig. S4B, the community heatmap analysis revealed the changes of the order, including the decrease of Bacilli, Saccharimonadia, Coriobacteria, Alphaproteobacteria, Actinobacteria, Cyanobacteria and Gammaproteobacteria, as well as the increase of Desulfovibrionia, Deferribacteres, Campylobacteria, Clostridia and Bacteroidia. In Fig. S4C, the bar plot showed the increase of Clostridia, Bacteroidia, Deferribacteres, and the decrease of Bacilli, Saccharimonadia, Coribacteria and Alphaproteobacteria. The Kruskal-Wallis H test bar plot in Fig. 4C analyzed the significance of differences at the class level, in which the abundance of Clostridia, Deferribacteres and Campylobacteria were higher in older age group. And the abundance of Bacilli, Saccharimonadia, Coribacteria and Alphaproteobacteria decreased in KK_2W, KK_4W and KK_8W, when compared to KK_0W, with significant difference. Specifically, there was significant difference of the proportion of Clostridia in KK_2W, KK_4W, and KK_8W group, when compared to KK_0W. Futhermore, microbiota alternation at the order level were detected. As illustrated in Fig. 4D, the relative abundance of Lachnospitales, Oscillospirales, Saccharimonadales, Deferribactrales and Campylobacterales was higher, and the relative abundance of Lactobacillales, Clostridia_UCG-014, Saccharimonadales, Erysipelotrichales, Coriobacteriales, RF39, Sphingomonadales, and Clostridiales decreased in KK_2W, KK_4W, and KK_8W group, when compared to KK_0W, with *p*<0.05.

**Fig. 5:**
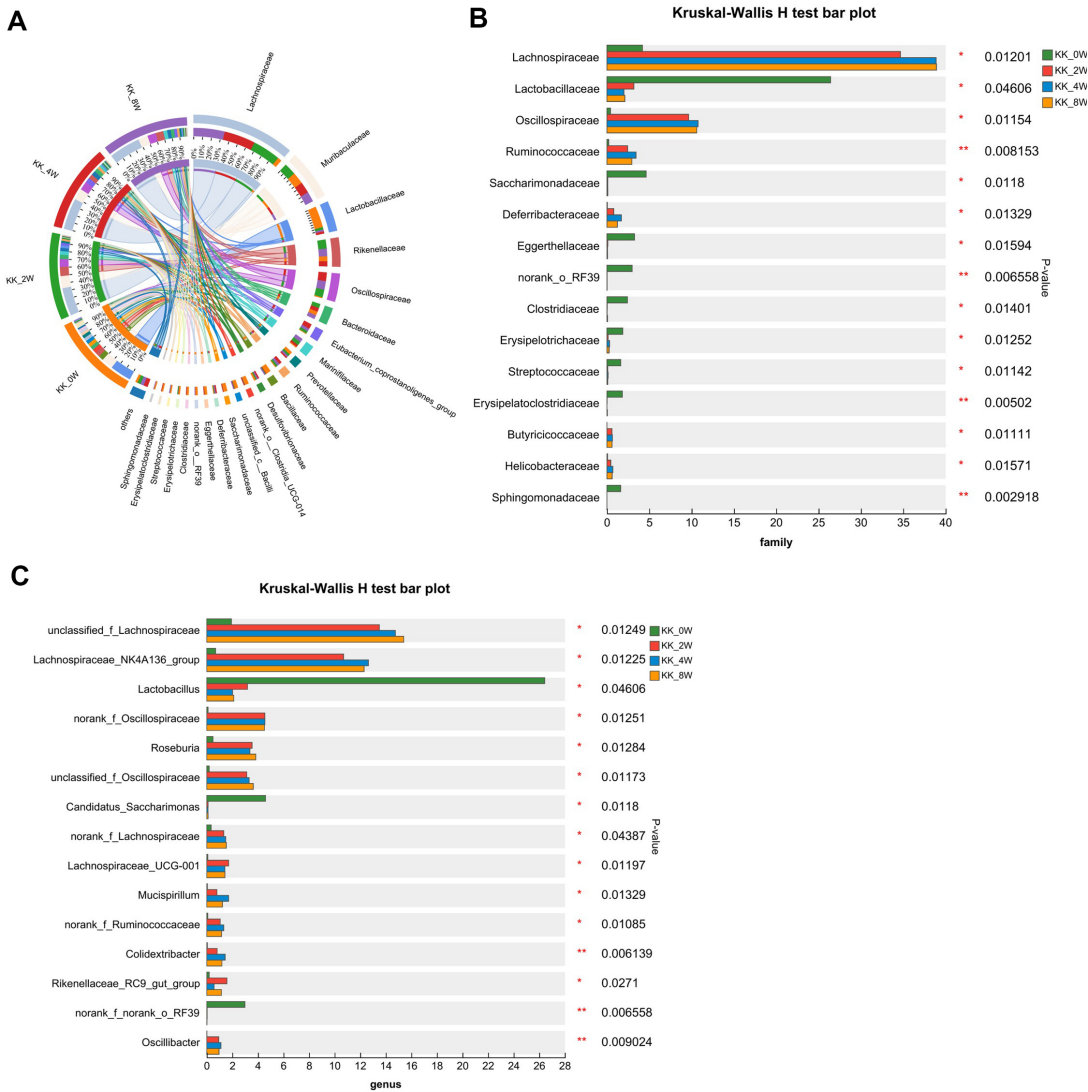
Gut microbiota compositions at the family and genus levels in KK-Ay mice of different ages. (**A**) Circos analysis depicting the gut microbiota composition at the family level; (**B, C**) Kruskal-Wallis H test bar plot revealing significant differences at the family and genus level, respectively. KK_0W, KK-Ay mice at 10 weeks old; KK_2W, KK-Ay mice at 12 weeks old; KK_4W, KK-Ay mice at 14 weeks old; KK_8W, KK-Ay mice at 18 weeks old; *p < 0.05, between groups; **p < 0.01, between groups.

At the family level, the Circos analysis in Fig. 5A and bar plot in Fig. S5A indicated the increase of Lachnospiraceae, Rikenellaceae, Oscillospiracceae, Eubacterium_coprostanoligenes_group, Marinifilaceae, and Ruminococcaceae, as wells as decrease of the abundance of Lactobacillaceae, Prevotellaceae and Bacillaceae. Kruskal-Wallis H test bar plot in Fig. 5B further demonstrated the significant increase in the abundance of Lachnospiraceae, Butyricicoccaceae, Oscillospiracceae, Deferribacteraceae, Ruminococcaceae and Helicobacteraceae, decrease of the abundance of Lactobacillaceae, Saccharimonadaceae, Eggerthellaceae, Clostridiaceae, Erysipelotrichaceae, Streptococcaceae and Sphingomonadaceae. The heatmap in Fig. S5B indicated the changes of microbial composition at the genus level, and the significant difference were further calculated in Kruskal-Wallis H test in Fig. 5C. The results showed a significant higher abundance of genera *Lachnospiraceae_NK4A136_group*, *Roseburia*, *Colidextribacter*, *Rikenellaceae_RC9_gut_group*, and *Oscillibacter*, and lower abundance of *Lactobacillus* and *Candidatus_Saccharimonas* in KK_2W, KK_4W, and KK_8W group, when compared to KK_0W. Fig. S5C showed the Kruskal-Wallis H test bar plot analysis at the species level of KK-Ay mice with different age.

### The Gut Microbial Diversity of Antibiotic-Treated Model

**Fig. 6:**
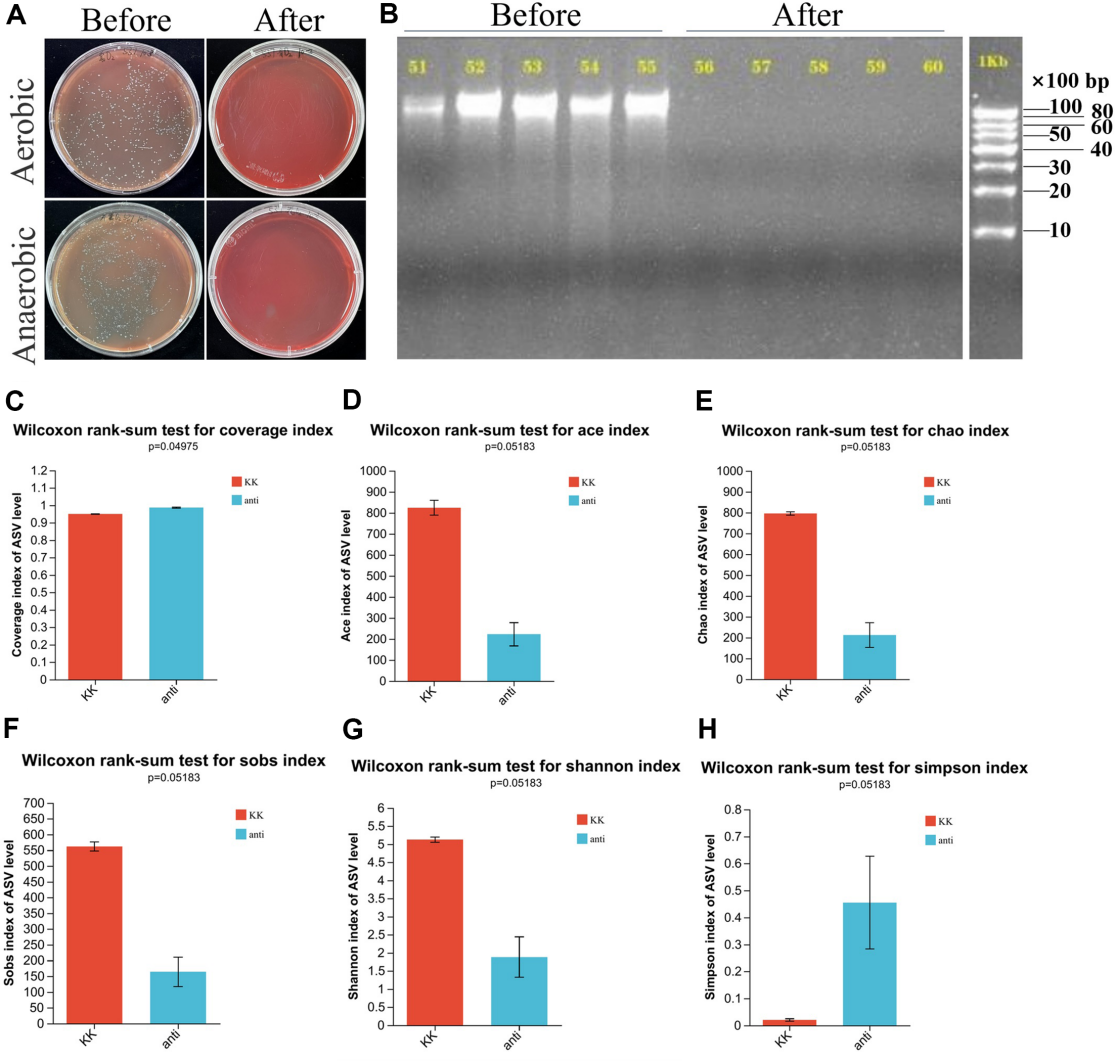
Analysis of fecal microbiota in antibiotic-treated KK-Ay mice. (**A**) Aerobic and anaerobic cultivation of intestinal contents from KK-Ay mice before and after antibiotic-treated treatment; (**B**) Agarose gel electrophoresis analysis of DNA abundance in KK-Ay mice with and without antibiotic-treated treatment; (**C**) Assessment of community diversity based on the Coverage metric; (**D**) Comparison of fecal microbial richness using the ACE index; (**E**) Calculation of fecal microbial richness using the Chao index; (**F**) Estimation of community diversitybased on the Sobs metric; (**G**) Evaluation of community diversity using the Shannon index; (**H**) Quantification of community diversity using the Simpson index. KK, KK-Ay group; anti: antibiotic-treated group.

To evaluate the efficacy of the antibiotic-treated model, colonic contents were collected from mice before and after antibiotic intervention. Subsequently, aerobic and anaerobic cultivation was conducted to determine the bacterial count per unit mass. The results in Fig. 6A demonstrated a significant decrease in bacterial count after intervention compared to before, indicating a total removal rate of up to 99.99% (data were attached in Table S1 and Table S2). Additionally, DNA extraction from colonic contents was performed for agarose gel electrophoresis. As illustrated in Fig. 6B, a noticeable decrease in DNA quantity was observed after antibiotic intervention.

Moreover, based on the data presented in Fig. 6C to 6H, comparable coverage was observed between the two groups. Following antibiotic intervention, a decline in fecal alpha-microbial richness was observed as determined by the ACE, Chao, and Sobs indexes. The diversity of the gut microbiome, as indicated by the Shannon index, decreased after antibiotic intervention, while the Simpson index showed an increase.

### The Effect of *R. gnavus* on Renal Function

**Fig. 7:**
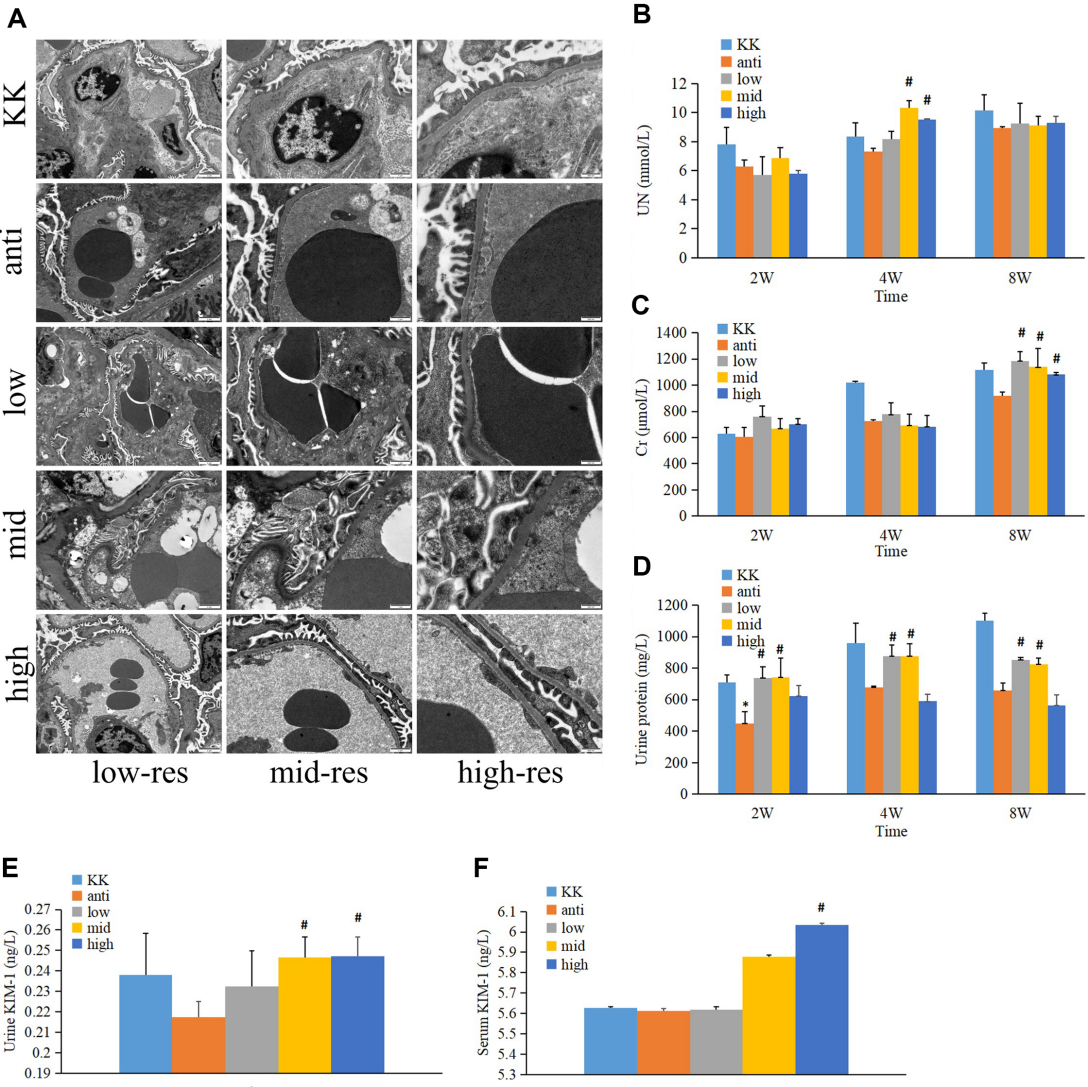
Impact of *R. gnavus* on kidney function. (**A**) Electron microscopy visualization of kidney; (**B**) Alterations in urine UN levels; (**C**) Changes in urine Cr levels; (**D**) Fluctuations in urine protein concentrations; (**E**) Variation in urine KIM- 1 levels; (**F**) Modulation of serum KIM-1 levels. KK, KK-Ay group; anti, antibiotic-treated group; low, low dose *R. gnavus* group; mid, middle dose *R. gnavus* group; high, high dose *R. gnavus* group; low-res, low resolution; mid-res, mid resolution; high-res, high resolution; *p < 0.05, v.s. KK-Ay group; ^#^*p* < 0.05, v.s. antibiotic-treated group.

To evaluate the impact of *R. gnavus* on renal function, kidney samples were collected for electron microscopy examination after sacrificing the animals. As shown in Fig. 7A, the glomeruli of the KK group exhibited widespread fusion of podocyte foot processes. However, antibiotic-treated treatment alleviated the fusion of podocytes. Upon *R. gnavus* treatment, the condition of podocyte was more severe in the low-dose group compared to the antibiotic-treated group, but similar to the KK group. Moreover, middle and high-dose *R. gnavus* treatment significantly increased the degree of podocyte fusion compared to the antibiotic-treated group, displaying a more severe phenotype than the KK group.

UN, Cr and urine protein serve as markers for kidney function assessment. In Fig. 7B, no significant differences were observed in UN level between the antibiotic-treated and KK groups after two weeks of treatment, although the UN level in the antibiotic-treated group appeared lower. Following *R. gnavus* administration, no significant differences in UN levels were found between the *R. gnavus* treatment group and the antibiotic-treated group. Similar trends were observed at eight weeks. However, after four weeks of treatment, the middle and high-dose groups showed significantly higher UN levels compared to the antibiotic-treated group.

Fig. 7C illustrated the changes in urine Cr levels after *R. gnavus* treatment. No significant differences were detected among the five groups after two weeks of treatment. Interestingly, the Cr level in the antibiotic-treated group was lower than in the KK group after four weeks of treatment. After eight weeks, the low, middle, and high-dose *R. gnavus* groups displayed significantly elevated Cr levels compared to the antibiotic-treated group.

The concentration of urine protein, depicted in Fig. 7D, exhibited a time-dependent increase in the KK group. Although not statistically significant, the urine protein levels in the antibiotic-treated group were lower than in the KK group. After two, four and eight weeks of treatment, the low and middle *R. gnavus* groups showed significantly higher urine protein levels compared to the antibiotic-treated group.

To confirm the influence of *R. gnavus* on kidney function, KIM-1 levels in urine and serum were measured using an ELISA kit. Fig. 7E and 7F revealed that after eight weeks of treatment, the middle and high-dose *R. gnavus* groups exhibited elevated urine KIM-1 levels, whereas the high-dose *R. gnavus* group showed increased serum KIM-1 levels, both significantly different from the antibiotic-treated group.

### The Effect of *R. gnavus* on Colon

**Fig. 8:**
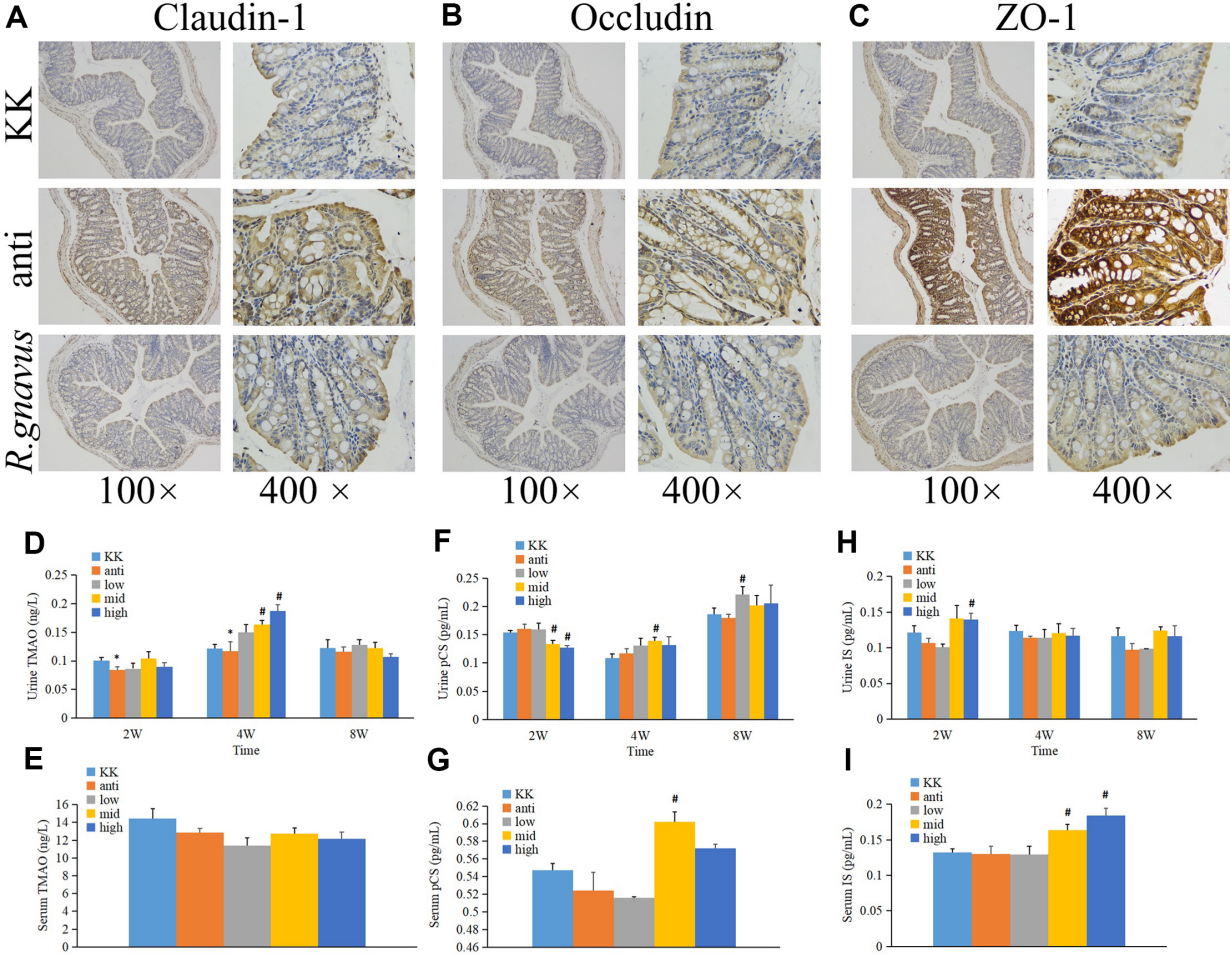
The effect of *R. gnavus* on colon. (**A**) Immunohistochemistry staining of Claudin-1 in colon; (**B**) Immunohistochemistry staining of Occludin in colon; (**C**) Immunohistochemistry staining of ZO-1 in colon; (**D**) Alternation of TMAO in urine; (**E**) Alternation of TMAO in serum; (**F**) Alternation of pCS in urine; (**G**) Alternation of pCS in serum; (**H**) Alternation of IS in urine; (**I**) Alternation of IS in serum. KK, KK-Ay group; anti: antibiotic-treated group; *R. gnavus*: *R. gnavus* treatment group; low, low dose *R. gnavus* group; mid, middle dose *R. gnavus* group; high, high dose *R. gnavus* group; *p < 0.05, v.s. KK-Ay group; #*p* < 0.05, v.s. antibiotic-treated group.

To assess the impact of *R. gnavus* on the colon, colon samples were collected for IHC staining. Fig. 8A to 8C illustrated the up-regulation of Claudin-1, Occludin, and ZO-1 expression after antibiotic-treated treatment, which was mitigated following *R. gnavus* administration. Additionally, we examined the concentration of uremic toxins, including TMAO, pCS, and IS, in urine and serum. In Fig. 8D, urine TMAO levels significantly decreased after two and four weeks of treatment compared to the KK group. However, after four weeks of treatment, middle and high doses of *R. gnavus* led to elevated urine TMAO levels compared to the antibiotic-treated group, indicating a significant difference. No significant changes in urine and serum TMAO levels were observed after eight weeks of treatment (Fig. 8E).

Regarding urine pCS levels (Fig. 8F), they were found to be lower after two weeks of middle or high dose *R. gnavus* treatment compared to the antibiotic-treated group (*p*<0.05). However, after four and eight weeks of treatment, the middle and low dose groups exhibited significantly higher pCS concentrations in urine, respectively. Furthermore, serum pCS levels were higher after eight weeks of middle dose *R. gnavus* treatment (Fig. 8G).

In Fig. 8H, uric IS levels demonstrated a significant increase after two weeks of high-dose *R. gnavus* treatment. However, no significant differences were observed in urine IS levels after four or eight weeks of *R. gnavus* treatment. Nevertheless, as depicted in Fig. 8I, after eight weeks of treatment, serum IS levels were significantly higher in the middle and high dose *R. gnavus* treatment groups compared to the antibiotic-treated group.

### The Effect of *R. gnavus* on Inflammation

**Fig. 9:**
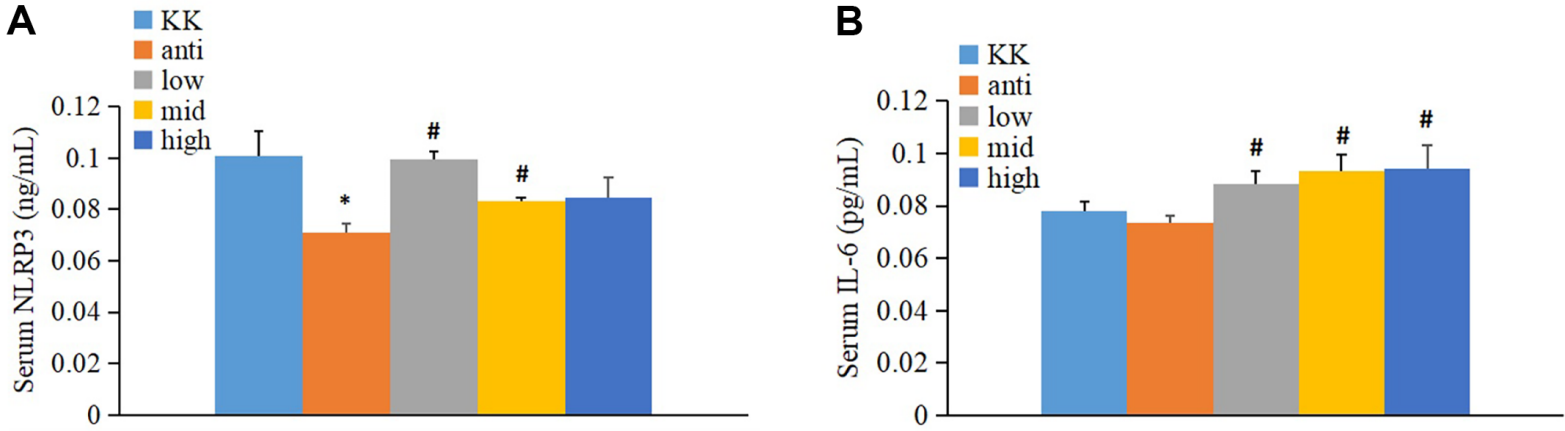
The effect of *R. gnavus* on inflammation. (**A**) Alternation of NLRP3 in serum; (**B**) Alternation of IL-6 in serum. KK, KK-Ay group; anti: antibiotic-treated group; low, low dose *R. gnavus* group; mid, middle dose *R. gnavus* group; high, high dose *R. gnavus* group; *p < 0.05, v.s. KK-Ay group; ^#^*p* < 0.05, v.s. antibiotic-treated group.

Upon completing the eight-week treatment, serum samples were obtained for analysis. Fig. 9A clearly illustrated that serum NLRP3 levels were down-regulated in the antibiotic-treated group compared to the KK group, with a significant difference (*p* < 0.05). However, treatment with low and middle doses of *R. gnavus* resulted in significantly higher serum NLRP3 levels compared to the antibiotic-treated group. Furthermore, Fig. 9B demonstrated a noteworthy elevation in serum IL-6 levels following *R. gnavus* administration when compared to the antibiotic-treated group.

## Discussion

Findings in this study present valuable insights into how DN is associated with changes in gut microbiota composition. The study shows a significant increase in the abundance of Clostridia at the class level, higher levels of Lachnospirales and Oscillospirales at the order level, and a notable decrease of Clostridia_UCG-014. Additionally, there is a noteworthy increase in the abundance of Lachnospiraceae, Oscillospiraceae, and Ruminococcaceae at the family level. These changes are observed in relation to both the initiation and progression of DN.

Clostridia belong to the phylum Firmicutes, and this study's findings are consistent with a research by Randall *et al*. [35]. They discovered differences in microbial population dynamics in animals with kidney disease, such as increased alpha diversity, relative decreases in Lachnospiraceae and *Lactobacillus*, and increases in some Clostridia and opportunistic taxa. Although few studies have directly linked Clostridium infection to type 1 diabetes mellitus (T1DM) initiation [36, 37], many studies have suggested that disturbance of Clostridia is related to glucose dysregulation in patients [38-44]. Our study is among the few to explore the abundance of Clostridia at the class level in DN. We found that Lachnospirales at the order level and Lachnospiraceae at the family level were more abundant in DN. Similar results were also observed in subjects with gestational diabetes mellitus (GDM), indicating an enriched bacterial operational taxonomic unit in the family Lachnospiraceae [45]. However, a meta-analysis of 16 studies found that the Lachnospiraceae family was depleted in DN patients compared to healthy controls [46]. Our study was the first to discuss the abundance of Oscillospirales at the order level and Oscillospiraceae at the family level. We also noted a higher abundance of the Ruminococcaceae family in DN patients. Similar results were found in patients with GDM, where glucose levels were positively correlated with the Ruminococcaceae family [46]. We found that Clostridia_UCG-014 was down-regulated in DN, but it was strongly positively correlated with fasting blood glucose in type 2 diabetic Goto-Kakizaki rats [48]. The relationship between gut microbiota and DN remains controversial and requires more research to deepen our understanding.

In 1976, Moore *et al*. initially identified *R. gnavus* as a significant component of the human gut microbiota. It was initially classified as part of the genus *Ruminococcus* within the family Ruminococcaceae. However, further analysis using 16S rRNA gene sequencing led to its reclassification as a species belonging to the Firmicutes phylum, Clostridia class, Clostridium cluster XIVa, and Lachnospiraceae family [49]. The abundance imbalance of *R. gnavus*, leading to gut dysbiosis, has been suggested as a possible risk factor for the development of inflammatory and metabolic diseases. To investigate this, we conducted a study using antibiotic-treated mice with DN. We found that orally administering *R. gnavus* to these mice accelerated the progression of DN-related symptoms. Specifically, we observed pathological changes in the kidneys, as well as increased levels of UN, Cr, and urine protein. Interestingly, the presence of *R. gnavus* in the gut has been found to impact the expression of proteins responsible for maintaining the integrity of the intestinal barrier. This can result in increased permeability and disruption of the barrier function. Additionally, elevated levels of uremic toxins, such as TMAO, pCS, and IS, have been observed in urine and serum samples. These findings suggest that the gut-kidney axis plays a significant role in DN [50]. There is growing evidence supporting the notion that the interaction between the host and the gut microbiota is relevant in the development and progression of CKD, including DN. This interaction is bidirectional, with uremia affecting the composition and metabolism of the gut microbiota, while microbial metabolism contributes to the production of important uremic toxins. Dysbiosis of the gut microbiota can compromise the intestinal barrier, allowing increased exposure to endotoxins [50]. In the context of CKD, alterations in diet and gastrointestinal function shift microbial metabolism towards the fermentation of protein sources. This leads to the generation of uremic toxins like IS and pCS, while TMAO is produced through microbial metabolism of choline and carnitine. The vascular and renal toxicity of these co-metabolites has been extensively demonstrated in experimental and clinical studies, rendering them an appealing target for adjuvant therapy in DN [50].

Furthermore, our research indicates that oral administration of *R. gnavus* induces the up-regulation of inflammatory factors such as NLRP3 and IL-6. Dysbiosis of the gut microbiota can result in the translocation of endotoxins and pathogens across the intestinal barrier, triggering inflammation and oxidative stress, which further contribute to renal damage [51]. In addition, gut microbial dysbiosis may lead to the alteration of microbial metabolites, which are considered important substances that regulate life activity and metabolism and participate in the onset and progression of various diseases [23].

## Conclusion

In general, our findings strongly indicate that administering *R. gnavus* exacerbates DN by influencing the levels of uremic toxins and promoting inflammation in antibiotic-treated DN cases.

## Supplemental Materials

Supplementary data for this paper are available on-line only at http://jmb.or.kr.
