# Supplementary material for: Specific Alternation of Gut Microbiota and the Role of Ruminococcus gnavus in the Development of Diabetic Nephropathy
Source: J Microbiol Biotechnol. 2023 Dec 26;34(3):547–61. doi: 10.4014/jmb.2310.10028 (PMC11016775; doi:10.4014/jmb.2310.10028)
Supplement: Supplementary file 1 [file jmb-34-3-547-supple.pdf]

J. Microbiol. Biotechnol.
https://doi.org/10.4014/jmb.2310.10028

Supplementary Figures

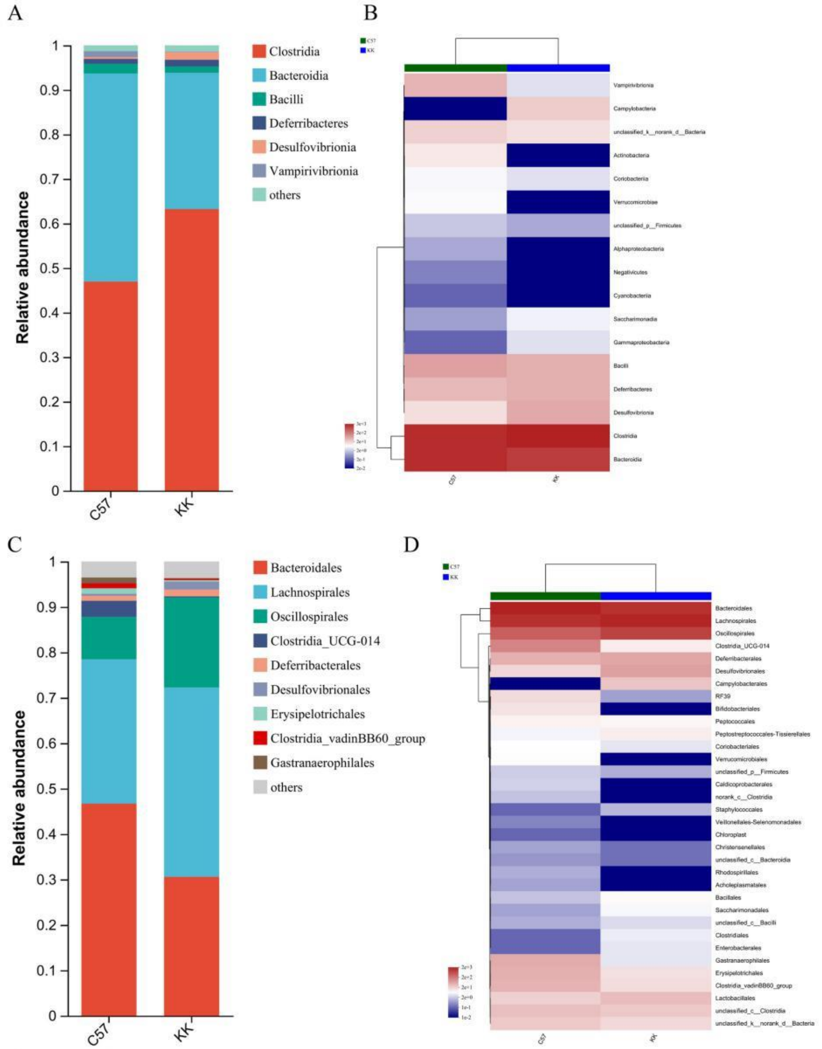

Supplementary Figure 1 Gut microbiota compositions ranging from class to order levels in C57 and KK-Ay mice. A. Community bar plot analysis illustrating the distribution of microbial classes in the gut microbiota; B. Community heatmap analysis depicting the variation in class-level gut microbiota composition; C. Community bar plot analysis displaying the abundance of different orders in the gut microbiota; D. Community heatmap analysis indicating the patterns of order-level gut microbiota composition.

Supplementary Fig. S1

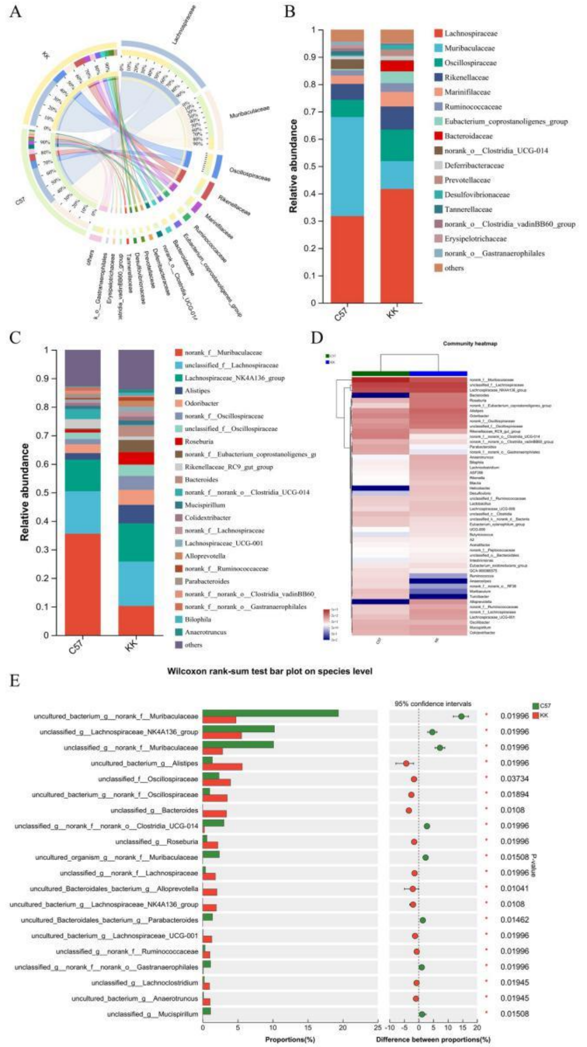

Supplementary Figure 2 Analysis of gut microbiota compositions at the family to species levels in C57 and KK-Ay Mice. A. Circos analysis displaying the gut microbiota composition at the family level; B-C. Community bar plot analysis presenting the gut microbiota composition at the family and genus level, respectively; D. Community heatmap analysis visualizing the gut microbiota composition at the genus level; E. Wilxocon rank-sum test bar plot at the species level. C57, C57BL/6J group; KK, KK-Ay group; *P<0.05, v.s. C57BL/6J group.

Supplementary Fig. S2

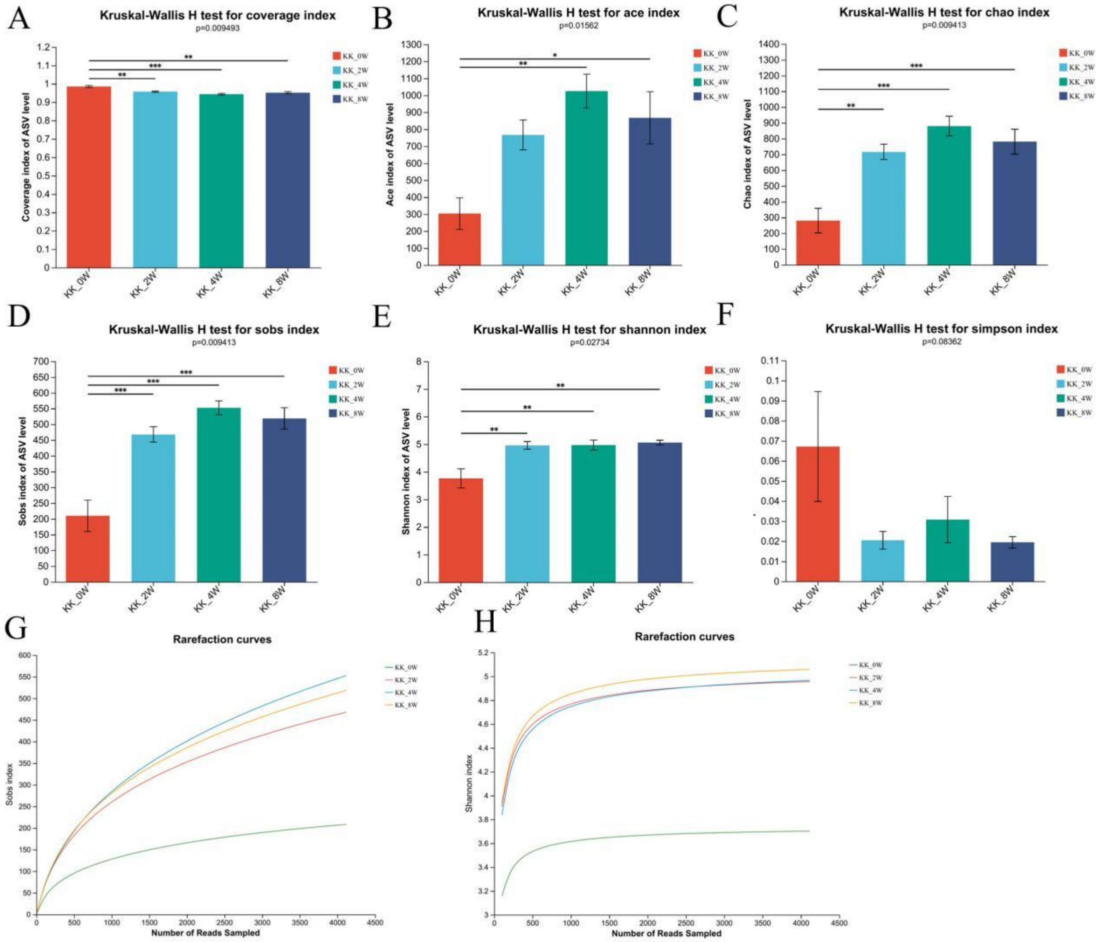

Supplementary Figure 3 The fecal microbiota analysis of KK-Ay mice with different age. A. The community diversity calculated by Coverage; B. The fecal microbial richness calculated by ACE; C. The fecal microbial richness calculated by Chao; D. The community diversity calculated by Sobs; E. The community diversity calculated by Shannon index; F. The community diversity calculated by Simpson index; G. The rarefaction curves of Sobs index; H. The rarefaction curves of Shannon index. KK_0W, KK-Ay mice at 10 weeks old; KK_2W, KK-Ay mice at 12 weeks old; KK_4W, KK-Ay mice at 14 weeks old; KK_8W, KK-Ay mice at 18 weeks old; $^{*}P<0.05$, between groups; $^{**}P<0.01$, between groups; $^{***}P<0.001$, between groups.

Supplementary Fig. S3

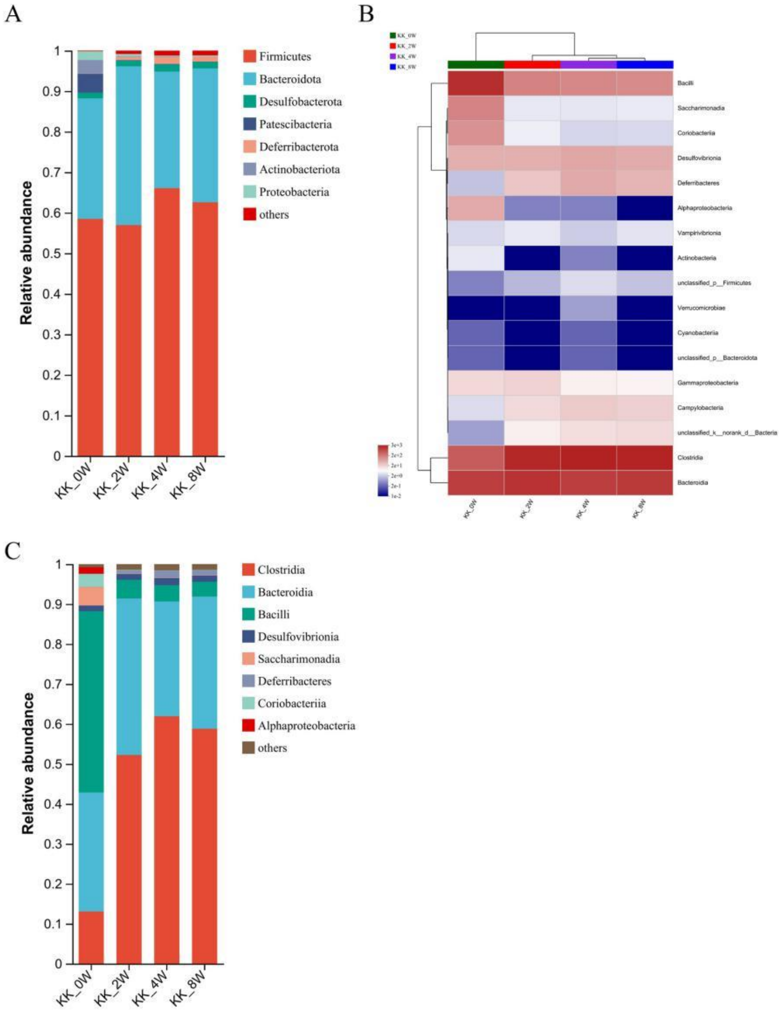

Supplementary Figure 4 Gut microbiota compositions ranging from phylum to order of KK-Ay mice with different age. A. The community bar plot analysis provides an overview of the gut microbiota composition at the phylum level; B. The community heatmap analysis reveals the gut microbiota composition at the class level; C. The community bar plot analysis depicts the relative abundance of gut microbiota at the class level. KK_0W, KK-Ay mice at 10 weeks old; KK_2W, KK-Ay mice at 12 weeks old; KK_4W, KK-Ay mice at 14 weeks old; KK_8W, KK-Ay mice at 18 weeks old; $^{*}P<0.05$, between groups; $^{**}P<0.01$, between groups.

Supplementary Fig. S4

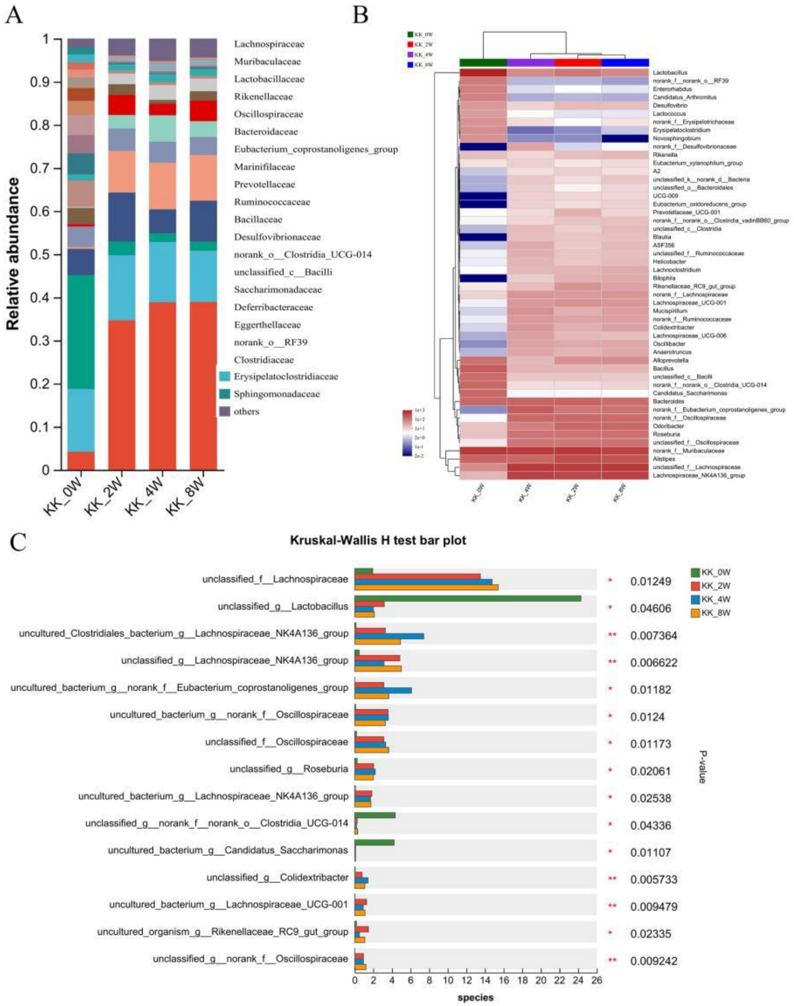

Supplementary Figure 5 Gut microbiota compositions ranging from the family to species levels in KK-Ay mice of different ages. A. Community bar plot analysis illustrating the gut microbiota composition at the family level; B. Community heatmap analysis displaying the gut microbiota composition at the genus level; C. Kruskal-Wallis H test bar plot at the species level. KK_0W, KK-Ay mice at 10 weeks old; KK_2W, KK-Ay mice at 12 weeks old; KK_4W, KK-Ay mice at 14 weeks old; KK_8W, KK-Ay mice at 18 weeks old; **P*<0.05, between groups; ***P*<0.01, between groups.

Supplementary Fig. S5

Supplementary Table S1. Bacterial count before germ-free intervention.

Group	Mouse NO.	Volum/mL	Dilution ratio	Bacterial count/CFU		Sample mass/g	Bacterial density per unit weight(CFU/g)
				After dilution	Before dilution		
Aerobic	536	1	1000	51	5.10E+05	0.0197	2.59E+07
	537	1	100000	208	2.08E+08	0.0235	8.85E+09
	545	1	1000	382	3.82E+06	0.0172	2.22E+08
Anaerobic	536	1	1000	76	7.60E+05	0.0197	3.86E+07
	537	1	100000	213	2.13E+08	0.0235	9.06E+09
	545	1	1000	414	4.14E+06	0.0172	2.41E+08
Amount							1.84E+10

Supplementary Table S2. Bacterial count after germ-free intervention.

Group	Mouse NO.	Volum/mL	Dilution ratio	Bacterial count/CFU		Sample mass/g	Bacterial density per unit weight(CFU/g)	Overall removal efficiency
				After dilution	Before dilution			
Aerobic	536	1	100	27	2.70E+04	0.2196	1.23E+05	0.999966278
	537	1	100	14	1.40E+04	0.1434	9.76E+04	
	545	1	100	12	1.20E+04	0.1565	7.67E+04	
Anaerobic	536	1	100	20	2.00E+04	0.2196	9.11E+04	
	537	1	100	17	1.70E+04	0.1434	1.19E+05	
	545	1	100	18	1.80E+04	0.1565	1.15E+05	
Amount							6.22E+05	
